# Decoding declarative memory process for predicting memory retrieval based on source localization

**DOI:** 10.1371/journal.pone.0274101

**Published:** 2022-09-08

**Authors:** Jenifer Kalafatovich, Minji Lee, Seong-Whan Lee

**Affiliations:** 1 Department of Artificial Intelligence, Korea University, Seoul, Republic of Korea; 2 Department of Brain and Cognitive Engineering, Korea University, Seoul, Republic of Korea; La Sapienza University of Rome, ITALY

## Abstract

Many studies have focused on understanding memory processes due to their importance in daily life. Differences in timing and power spectra of brain signals during encoding task have been linked to later remembered items and were recently used to predict memory retrieval performance. However, accuracies remain low when using non-invasive methods for acquiring brain signals, mainly due to the low spatial resolution. This study investigates the prediction of successful retrieval using estimated source activity corresponding either to cortical or subcortical structures through source localization. Electroencephalogram (EEG) signals were recorded while participants performed a declarative memory task. Frequency-time analysis was performed using signals from encoding and retrieval tasks to confirm the importance of neural oscillations and their relationship with later remembered and forgotten items. Significant differences in the power spectra between later remembered and forgotten items were found before and during the presentation of the stimulus in the encoding task. Source activity estimation revealed differences in the beta band power over the medial parietal and medial prefrontal areas prior to the presentation of the stimulus, and over the cuneus and lingual areas during the presentation of the stimulus. Additionally, there were significant differences during the stimuli presentation during the retrieval task. Prediction of later remembered items was performed using surface potentials and estimated source activity. The results showed that source localization increases classification performance compared to the one using surface potentials. These findings support the importance of incorporating spatial features of neural activity to improve the prediction of memory retrieval.

## Introduction

Memory processes have been widely studied due to their importance in daily life. Memory not only allows us to store information and retrieve it as required, but also to learn from past experiences to modify or guide our behavior [1]. Previous studies have concentrated on understanding brain mechanisms involved in memory processes, such as changes in brain activity and neural oscillations related to later remembered items [2, 3] and, more recently, in the prediction of remembered items in the retrieval task using information obtained prior and during the presentation of the stimulus (learning or encoding task) [4]. Prediction of later successfully remembered items can be used for enhancing learning efficiency; and early diagnosis and treating diseases that impair memory abilities [1]. Classifying which items are going to be remembered or forgotten can be simply seen as a binary classification problem [5]; however, it is more complicated than that because the information used for prediction and its results belong to different tasks. Therefore, it is important to extract relevant features from one task that reflects the performance of the other task.

Changes in brain activity between later remembered and forgotten items have been studied before [6]. These differences are present prior to and during the presentation of a stimulus. Differences in event-related potentials (ERP) were found between 400 and 800 ms during the presentation of the stimulus [7]. Additionally, a negative activity around 250 ms was found in frontal areas prior to stimulus onset [8]. Some studies showed that neural oscillation differences in different frequency bands are correlated with the later remembering and forgetting of a stimulus. Increased activity was found in the theta frequency band during pre-stimulus [9], especially in medial temporal areas [10]. Other studies showed that an increase in low beta power prior to the presentation of the stimulus was related to the ability to form new memory traces [2]. An increase in gamma band power related to remembered items was also found during the presentation of the stimulus in the encoding task [11]. In contrast, a decrease in alpha and beta band power after stimulus onset was found to be related to successfully remembered items [12]. Salari and Rose [13] found that an increase in theta and beta power was related to the likelihood of remembering the presented stimulus. These changes in power were applied to a brain-computer interface; the presentation of the stimulus was modulated by the participant’s state (a stimulus was either presented during a period of increase in theta or beta power). It was found the memory performance only increased when the stimulus was presented during a high beta state, suggesting that the increase in beta power is highly related to memory encoding and formation.

Many studies revealed that structures such as the hippocampus [14], thalamus [15], anterior cingulate, and medial prefrontal cortex [16, 17] and others contained relevant information for the formation of a memory. This phenomenon was observed through electrocorticography (ECoG) or functional magnetic resonance imaging (fMRI). However, their use involves invasive procedures or high cost, respectively. In contrast, electroencephalogram (EEG) is practical and more convenient to use due to its low cost and the fact that involves non-invasive procedures. EEG signals have been successfully used in many paradigms (e.g., motor imagery classification, cognitive state recognition, and sleep monitoring) [18–20], proving its feasibility.

Previous studies have explored the possibility of predicting later successfully remembered items in different memory paradigms such as working memory and declarative memory tasks [4, 21, 22]. Working memory retains and manipulates information over short periods of time (sec) [23]. Meanwhile, declarative memory can store information for later recall [24]. The difference between declarative memory and working memory tasks is reflected in the time between the encoding and retrieval tasks and the number of presented stimuli [25].

An EEG study combined temporal and spectral features for the prediction of successfully remembered images in a declarative memory paradigm [4]. Temporal features were extracted during the presentation of the stimulus and classified using linear discriminant analysis (LDA). Simultaneously, spectral features were extracted using the common spatial pattern (CSP) prior and during the presentation of the stimulus, and support vector machine (SVM) was used for classification. An average accuracy of 59.6% was obtained after combining the classifiers. In Höhne et al. [26], intracranial EEG data from the medial temporal lobe was analyzed to predict successful memory encoding. The experimental paradigm consisted of the presentation of 300 German nouns for 300 ms. They analyzed the significant phase clustering of different time windows and frequencies across participants; later they identified the time windows and frequencies for which the phases differed depending on the remembered and forgotten trials per participant. SVM was used as a classifier, an average classification accuracy of 69.2% was obtained. As observed, high accuracies were obtained in studies using ECoG [26]; however, EEG studies present a dramatic decrease in accuracy [4]. This phenomenon can be explained by the high noise-to-signal ratio characteristic of EEG and also by the fact that EEG signals measure cortical brain activity.

In this work, we propose a framework for predicting later remembered items during the encoding task of a declarative memory paradigm. In order to increase prediction accuracy, we estimated source activity through source localization and used it for classification. Estimated source activity can provide the spatial location of the measured signal. Signals were divided into theta (4–8 Hz), alpha (8–12 Hz), beta (12–30 Hz), and gamma (30–40 Hz) bands due to the importance of neural oscillations changes related to successfully remembered and forgotten stimuli [11, 27, 28]. We compared prediction accuracies when using surface potentials and estimated source activity data. We hypothesized that higher accuracy can be obtained when using estimated signals due to the use of reconstructed spatial features. Our framework could be applied in systems that attempt to increase memory abilities connected to memory-related diseases.

## Materials and methods

### Participants

Thirteen healthy participants were recruited to participate in the experiment (five women, 24–31 years of age). All had a normal or corrected-to-normal vision and no history of neurological disease. Participants had more than 10 years of English education; recruitment was performed among university students. Experiments were conducted according to the principles described in the Declaration of Helsinki. This study was reviewed and approved by the Institutional Review Board at Korea University (KUIRB-2019–0269-01). Written informed consent was obtained before the experiment.

### Experimental design

**Fig 1:**
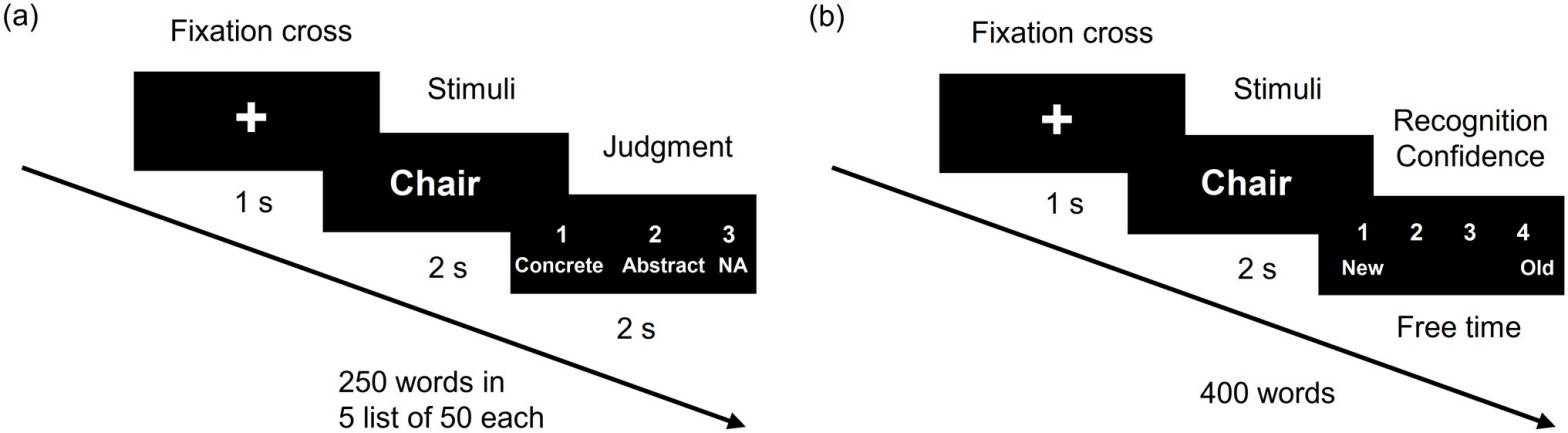
Experimental timeline of the declarative memory paradigm. (a) Encoding task: 250 words are presented in five lists of 50 words each. (b) Retrieval task: 400 words (250 old words and 150 new words) are presented. Participants have to select whether the presented stimulus is an old or new word, depending on the stimulus presented in the encoding task. These two tasks are separated by an arithmetic distraction task.

Fig 1 illustrates the experimental paradigm, which consisted of encoding and retrieval tasks. The experimental paradigm was implemented using Psychophysics Toolbox. All presented words were chosen randomly from a pool of the 3,000 most commonly used words according to Oxford University. In total, 250 words were shown during the encoding task (50% were concrete and 50% were abstract), divided into five lists of 50 words each. A black screen was presented between lists for 5,000 ms. A trial consisted of the presentation of the fixation cross on the monitor for 1,000 ms followed by a stimulus (a word, specifically an English noun) for 2,000 ms. Finally, participants were asked to choose, using the keyboard, if the presented word was abstract (e.g., happiness, anger) or concrete (e.g., paint, house) or NA (subjects can select these options whenever they are not sure if the presented word was abstract or concrete) within 2,000 ms after which the next trial started. Semantic judgment of words was shown high memorability compared to other judgments or the words presented without any judgment [26]. The total duration of the encoding task was 21 m.

During the retrieval task, 400 words were presented, in which 250 words presented in the encoding task and 150 new words were included. A trial consisted of the presentation of a fixation cross for 1,000 ms followed by the presentation of the stimulus for 2,000 ms. Finally, participants were asked to select a recognition confidence level from 1 to 4 (1: new word, 2: maybe new, 3: maybe old, 4: old word—for classification purposes, new word and maybe new were chosen, it was considered that the subject perceived the presented word as new, same was considered for old words). There was no time limit for selecting the recognition confidence; therefore, the duration of the retrieval task varied among participants (average time: 27 m). Encoding and retrieval tasks were separated by a distraction task. Participants were asked to count backward from 1,000 to zero in steps of seven for 20 m. This aims to prevent the rehearsal of previously presented words.

### Data acquisition and preprocessing

Data were recorded from 62 Ag/AgCl electrodes applied to the scalp using Brain Vision/Recorder (BrainProduct GmbH, Germany). Electrodes were placed following the international 10–20 system with a sampling rate of 1,000 Hz. Reference and ground electrodes were placed at the FCz and FPz positions, respectively. Prior experiment, all electrode impedances were measured and set below 10 *kΩ* using EEG gel. Participants sat in a comfortable chair, facing a 19-inch liquid crystal display monitor.

EEG signals were down-sampled to 250 Hz and band-pass filtered using a fifth-order Butterworth filter from 0.5 to 40 Hz. These were performed to reduce computational time and reduce the noise-to-signal ratio, respectively. Data was re-referenced to the average of all channels. For encoding epochs, data were separated into later remembered (selected 3: maybe old or 4: old in the retrieval task), forgotten items (selected 1: new word or 2: maybe new in the retrieval task). For decoding task, data were separated into remembered (words that appeared during the encoding phase and were labeled as maybe old or old by the subject during the retrieval task); forgotten items (words that appeared during the encoding phase and were labeled as a new word or maybe new by the subject during the retrieval task); and false remembered items (words that did not appear during the encoding task but were labeled as maybe old or old by the subject during the retrieval task). Trials were epoched in relation to stimulus onset in intervals of 1,000 ms prior stimulus (pre-stimulus) and 1,000 ms during stimulus (on-going stimulus). All epochs were baseline corrected over the whole period. Channels were interpolated using the spherical method and epochs were rejected when the amplitude value exceeded a threshold of ± 200 μV, this was done to remove artifacts from the muscles and eye movements. After trial rejection, a total of 2395 trials ranging from 40 to 100 trials per class were kept and analyzed. Data were preprocessed using MATLAB and EEGLAB toolbox [29].

### Feature extraction

Feature extraction was performed for surface potentials and source activity. For surface potentials, we extract time-frequency features, while for source activity additional spatial features are computed.

#### Surface potentials

The signal processing was performed using the multitaper method. A Hanning window, which is a bell-shaped curve, was used as the taper, with a window length of 100 ms (overlap of 50%). Frequencies were chosen from 4 to 40 Hz in bins of 1 Hz.

$$\begin{array}{c} S_{xj}^{L,k}={\left|FT_{W_{k}^{L}xj}\left(f\right)\right|}^{2}/F_{s},f\in \left[0,F_{s}\right]Hz \end{array}$$ (1)

where $FT_{\left(W_{k}^{L}xj\right)}\left(f\right)$ is the Fourier transform of $W_{k}^{L}xj$, which defined the window. Finally, power was averaged in theta (4–8 Hz), alpha (8–12 Hz), beta (12–30 Hz), and gamma (30–40 Hz) bands due to their relevance to memory processes [13]. Delta activity is not directly related to memory processes [30]; therefore, we excluded it from the analysis. This analysis was performed over pre-stimulus and on-going stimulus segments for encoding task and during the presentation of the stimulus for the retrieval task.

#### Source activity

Source activity was estimated using Brainstorm software [31]. First, EEGLAB files were imported into the Brainstorm environment. A head model was then computed using OpenMEEG BEM software [32] and EEG channel information (a file with the electrode location was shared for all participants and corrected to fit Brainstorm anatomy). The minimum norm imaging method was used to estimate the sources. It finds the cortical current that fits the data through the forward model, which requires the noise and source covariance matrix. Therefore, the noise covariance matrix of the EEG signal was calculated for the inverse estimation. The standardized low-resolution brain electromagnetic tomography (sLORETA) [33] was used to normalize the estimated current density at each source location, resulting in 15,002 voxels.

Time-frequency analysis of the estimated source activity was calculated using Brainstorm software. The power was averaged for theta, alpha, beta, and gamma bands as with surface potentials.

### Classification

**Fig 2:**
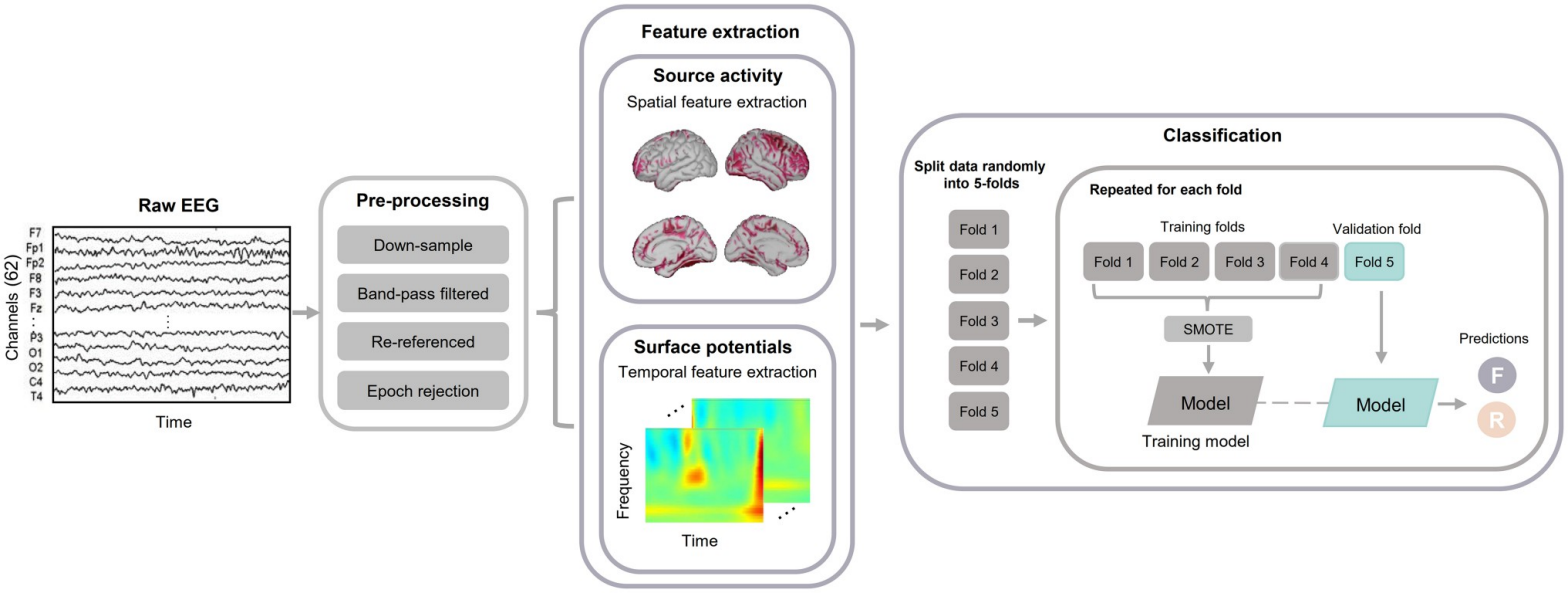
Signals analysis framework. Raw EEG data are preprocessed, after which source localization is conducted. Relevant features are extracted from surface potentials and estimated source activity, data is randomly split in five-folds. Four-folds are used as training data and the remaining one as validation data, this is repeated for each fold. The classifier aims to predict which items are going to be remembered or forgotten during the retrieval task in a declarative memory paradigm (F: forgotten items, R: remembered items).

As mentioned before, we tried to predict which items would be successfully remembered during the retrieval task, using signals prior to or during the presentation of the stimulus in the encoding task. Fig 2 shows the framework used; different classifiers were trained for each subject (subject-dependent classification). We reported macro-area under the receiver operation characteristic curve (AUC).

We trained different classifiers using the different feature extraction methods, to be specific we used shrinkage regularized linear discriminant analysis (rLDA) and a deep learning model. rLDA uses shrinkage to modify the covariance matrices, which can become an optimal classification method for high dimensional features. Covariance Σ is replaced by Σ(*γ*) = (1- *γ*) Σ + *γ* vI where *γ*∈ [0, 1] and is the tuning parameter; and v define the average of eigenvalues trace (Σ)/*d* of Σ (*d*: dimensionality of the feature space). The eigenvalue decomposition of Σ is define as follow: Σ = *VDV*^*T*^ with orthonormal V and diagonal D; then Σ(*γ*) = V((1-*γ*) D + *γ* vI) *V*^*T*^ [33]. Signals (channels × time, ♯channels = 62) are concatenated into a one-dimension vector along time points and input to the model. Deep learning has been applied successfully to different EEG studies [34–36]. We used a one-layer convolution neural network (CNN) and a fully connected layer as our deep learning model. The CNN layer used a kernel of (1, 3) with ELU as the activation function. Dropout (*p* = 0.25) and batch normalization were used at the output of the CNN layer. Adam was used to optimize all parameters, and cross-entropy was used as the loss function. The learning rate was set to 0.005 and the batch size to 25. The CNN model takes as input a two-dimension matrix (channels × time). Grid search was used to select hyper-parameters.

Prediction was performed using surface potentials and estimated source activity data. To evaluate the model, cross-validation was used. Cross-validation is widely used in EEG studies, its advantage is that all samples are used in training and validation, as a result, the variance of the estimated model performance is reduced [37]. Due to the data size, we used five-cross validation (80% is used as training set and 20% as validation set). Data were divided into five folds. Each fold was used as test data, while the remaining four were used to train the model. Samples were randomly assigned to a certain fold and the model was trained. Due to the nature of the task, subjects tend to remember more items than the forgotten ones, this results in a class imbalance. To address this problem, we used synthetic minority oversampling technique (SMOTE) over the training set and maintain the original test set. SMOTE has been used before to alleviate the class imbalance effects on the model performance [38], this method uses the feature space of the given data to generate synthetic samples. Results over the test set were averaged. In order the have a fair comparison between different feature selection methods, the same samples (trials) were used to train the model in each of the cases; even when the samples were chosen randomly, the use of “seeds” allowed us to reproduce the randomness.

### Statistical analysis

In the behavioral analysis, a paired *t*-test was used to investigate the difference between remembered and forgotten items associated with the selection of its nature (i.e., abstract or concrete). Differences in reaction times of remembered, forgotten, and falsely recognized items in the retrieval task (time that participants took to choose recognition confidence levels) were analyzed using repeated analysis of variance (ANOVA), and paired t-test was used as post-hoc. Reaction times during the encoding task (time that participants took for classifying a word as abstract or concrete) for remembered and forgotten items were also analyzed. Statistical analyses were performed in MATLAB. All significance levels were set to 0.05 with Bonferroni correction.

To evaluate the significant difference between remembered and forgotten activity in the different frequency bands, statistical analyses of surface potentials and estimated source activity were performed. To compare remembered and forgotten activity in different frequency bands, a parametric test (paired Student’s *t*-test) was performed. Due to the multiple comparisons (frequency × time), there is the possibility to observe increased rates of false positive cases; therefore, we implemented false discovery rate correction (Benjamini–Hochberg step-up procedure). To compare classification accuracy when using different feature extraction methods, a two-way repeated ANOVA was performed; one factor was frequency (theta, alpha, beta, and gamma bands), and the other factor was features (surface potentials or source activity). Paired *t*-test was used as a post-hoc test with Bonferroni correction. All significance levels were set to 0.05 with Bonferroni correction.

## Results

### Behavioral performance

Participants were able to remember on average 81.20 ± 9.00% of the items presented during the encoding task, and falsely recognized 20.80 ± 12.00% of the new words as previously presented items (false remembered items). During the encoding task, 94.76 ± 5.31% of the remembered items were classified either as abstract or concrete, while 82.58 ± 17.42% of the forgotten items were classified as either abstract or concrete. There was a significant difference between the number of items classified either as abstract or concrete for later remembered and forgotten items (*t*_(12)_ = -3.00, *p* = 0.012).

**Fig 3:**
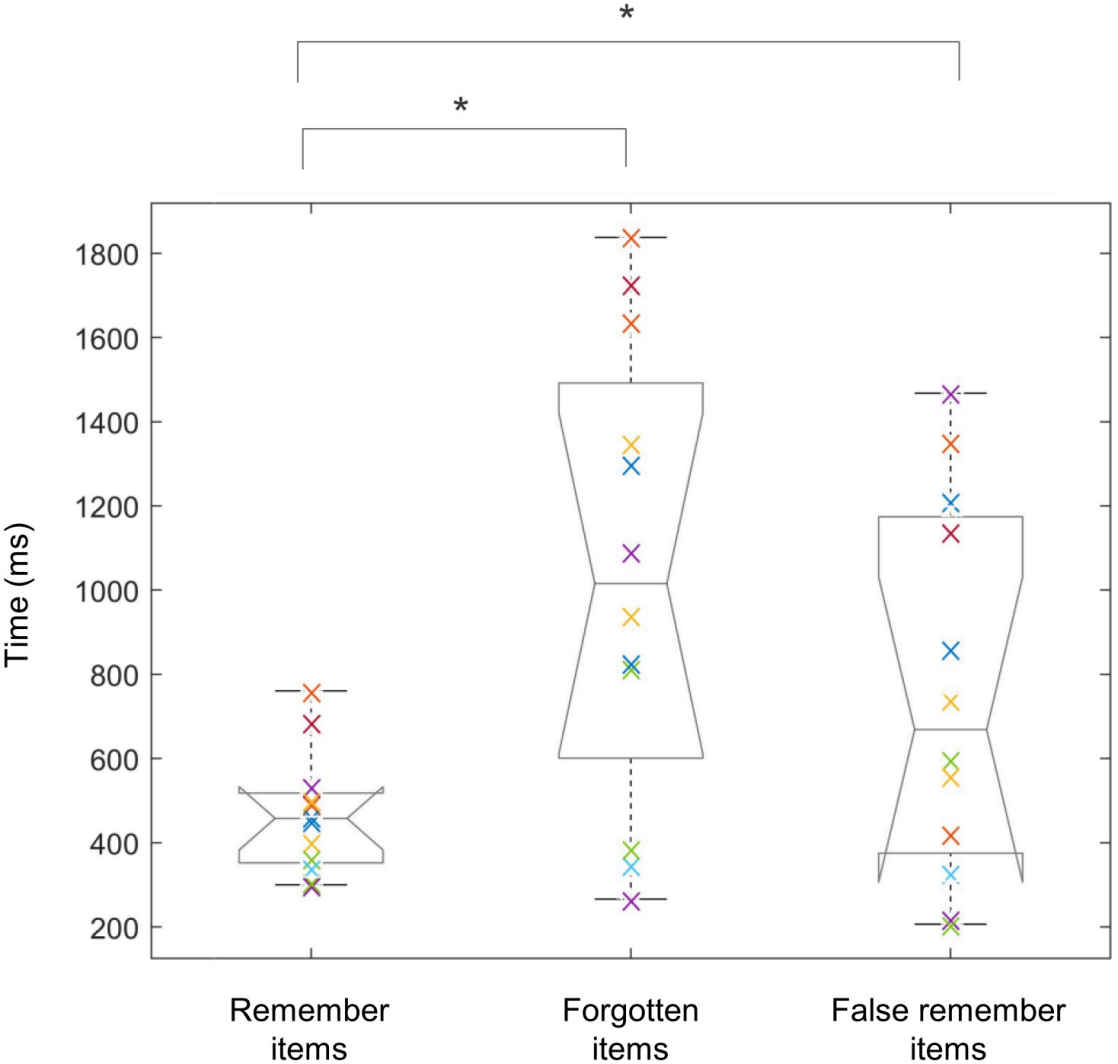
Reaction time of remember, forgotten, and false remember items for all participants and their mean value. Participants took more time to select the recognition confidence levels of forgotten words during the retrieval task. Boxplot indicates the mean, while the error bars indicate the standard deviation (* *p* < 0.05 with Bonferroni correction).

Reaction times in the encoding task (time that participants take to select if the word is concrete or abstract) were 784 ± 173 ms and 761 ± 160 ms for later remembered and forgotten items, respectively; no significant difference was found between reaction times in the encoding task (*t*_(12)_ = 1.086, *p* = 0.302). Reaction times in the retrieval task (time that participants take to select recognition confidence levels) were 483 ± 143 ms, 1223 ± 797 ms, and 736 ± 415 ms for remember, forgotten, and false remember items, respectively. Repeated ANOVA revealed significant differences between the reaction times (df = 2, F = 5.84, *p* = 0.006). Significant differences were found in reaction times for remember vs. forgotten items (*t*_(12)_ = -4.758, *p* < 0.001) and remember vs. false remember items (*t*_(12)_ = -2.89, *p* = 0.014) when performing post-hoc test (paired t-test). No significant difference was found for forgotten and false remember items (*t*_(12)_ = 2.32, *p* = 0.040). Fig 3 shows the reaction times in the retrieval task for all participants and the results of the statistical analyses.

### Differences for encoding task

ERP at Cz over all the subjects was calculated for encoding and decoding tasks (see S1(a) and S1(b) Fig, amplitude: *μ*V and time: ms). Statistical analyses revealed significant differences between remembered and forgotten items at different time points for Cz. High GFP (global field power) coefficients have been associated with a high signal-to-noise ratio [39]. GFP is calculated per conditions for encoding and decoding task following [40] since numbers of trials differ per condition. dGFP (difference GFP) wave is shown in S1(c) and S1(d) Fig along with the statistical results (amplitude: *μ*V and time: ms). High values of GFP were obtained and statistical differences were present at different time segments for both tasks.

#### Surface potentials

**Fig 4:**
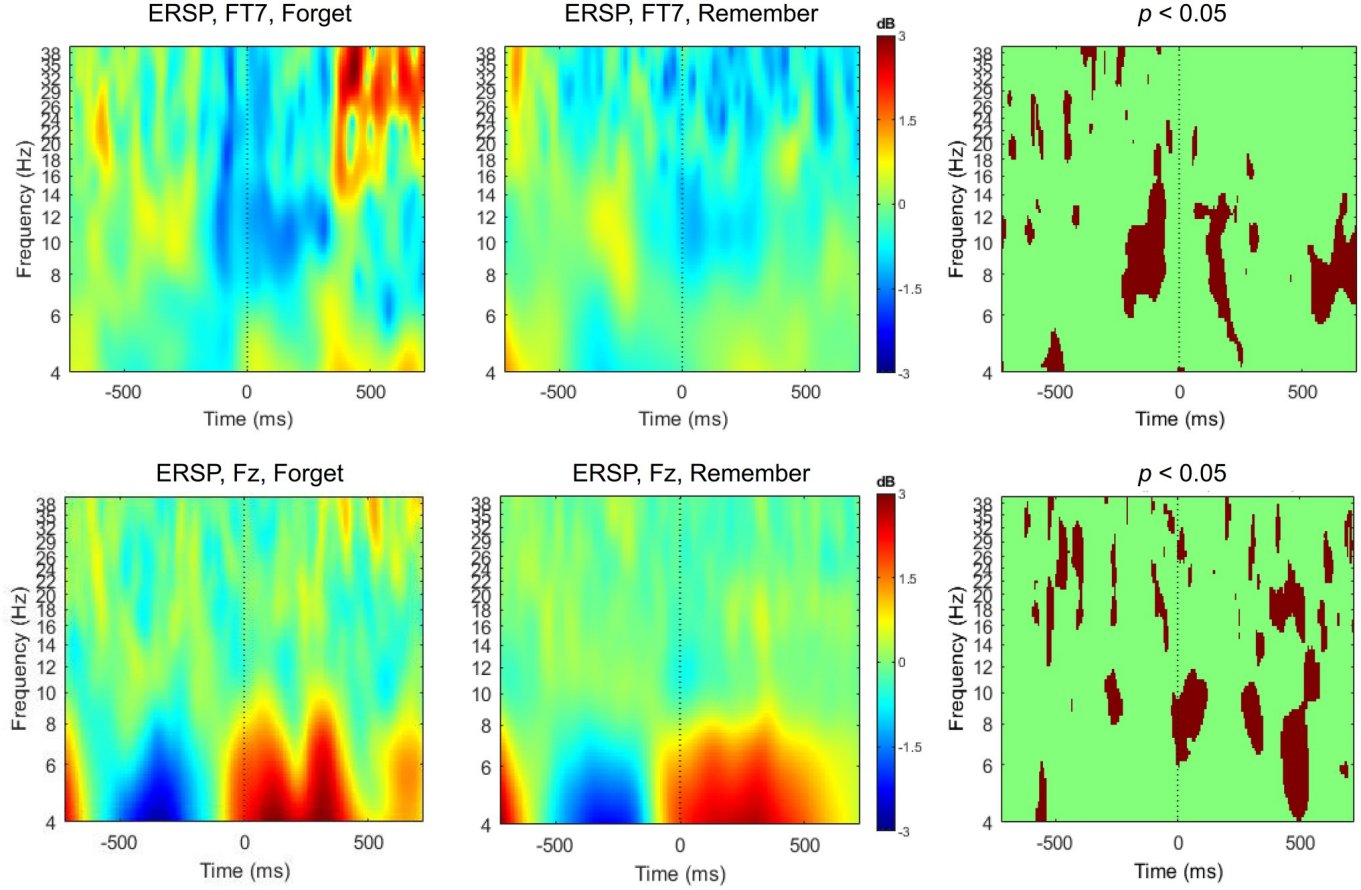
ERSP (event-related spectral perturbation) and statistical results of comparing surface potentials between later remembered items and forgotten items during encoding task for channel FT7 and Fz. Signals were separated per conditions and time-frequency analysis was performed. Statistical analysis revealed differences between conditions (* *p* < 0.05 with Bonferroni correction).

Statistical results of comparing later remembered and forgotten items are shown in Fig 4. Frequency-time analysis and statistical comparison revealed differences between conditions. For pre-stimulus segments there was a significant difference in the theta band over frontal region; in alpha and gamma band over right temporal region. For on-going stimulus, a significant difference was found in beta band over left temporal region. A frequency-time analysis revealed significant differences during -300–0 ms and 400–800 ms segments. As a result, these time windows were used for the analysis of reconstructed source activity.

#### Source activity

**Fig 5:**
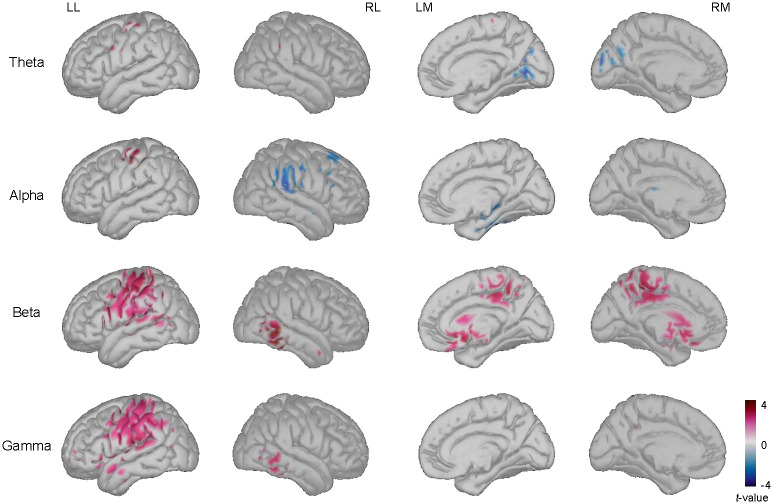
Significant difference between power of later remembered items vs. forgotten items during encoding task prior to the presentation of the stimulus. The *t*-values in theta, alpha, beta, and gamma bands when comparing remembered items to forgotten items during -300–0 ms prior to the presentation of the stimulus (two-tailed paired *t*-test, *p* < 0.05 with Bonferroni correction). LL = left lateral view; RL = right lateral view; LM = left medial view; RM = right medial view.

Reconstructed signals were averaged over -300–0 during pre-stimulus and statistical analysis was conducted. Fig 5 illustrates the statistical difference when comparing later remembered and forgotten items during -300–0 ms in the encoding task (pre-stimulus). For the encoding task, a decrease in the alpha band power was found for pre-stimulus segments related to later remembered items. Additionally, results showed an increase in the beta and gamma band power. Significant differences were found in the right parietal region for the alpha band power, and in the left parietal region for the beta and gamma band power. Differences in the right temporal region for the beta band power and in the left and right temporal regions for the gamma band power were found to be significant. Cuneus and lingual regions showed a significant decrease for the theta band power. Alpha band power showed a significant decrease in the parahippocampal regions. For beta and gamma band power, there was a significant increase in the medial parietal and medial prefrontal regions and an additional increase of the beta band power in the posterior cingulate and anterior cingulate.

**Fig 6:**
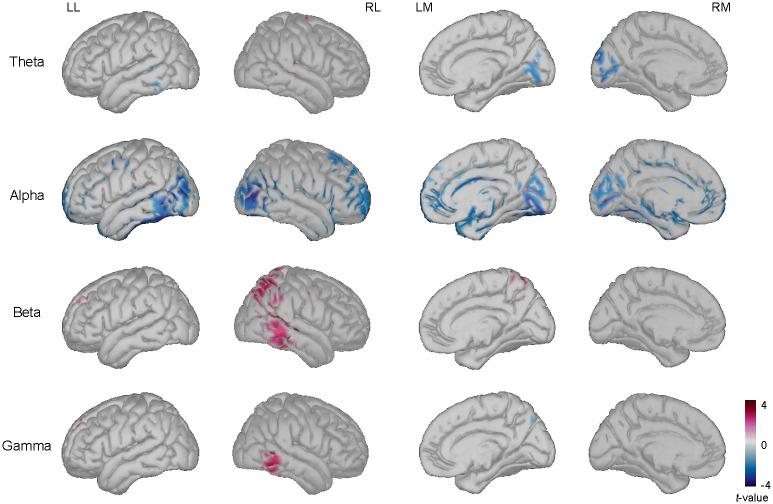
Significant difference between the power of later remembered items vs. forgotten items during stimulus presentation for encoding task. The *t*-values in theta, alpha, beta, and gamma bands when comparing the power in later remembered items and forgotten items at 400–800 ms during the presentation of the stimulus (two-tailed paired *t*-test, *p* < 0.05 with Bonferroni correction). LL = left lateral view; RL = right lateral view; LM = left medial view; RM = right medial view.

Reconstructed signals were averaged over 400–800 during on-going stimulus and statistical analysis was performed. Results showed a decrease of the alpha band power and an increase of the beta and gamma band power. Statistical results showed significant differences in the frontal, temporal, and occipital regions for the alpha band power. Additionally, there was a significant difference in the temporal regions for the beta and gamma band power, and in the parietal regions for the beta band power. Subcortical activity decreased for theta band and alpha band power; specifically, in the cuneus and lingual regions. Fig 6 depicts the statistical results when comparing later remembered and forgotten items in the encoding task during 400–800 ms.

### Differences for retrieval task

**Fig 7:**
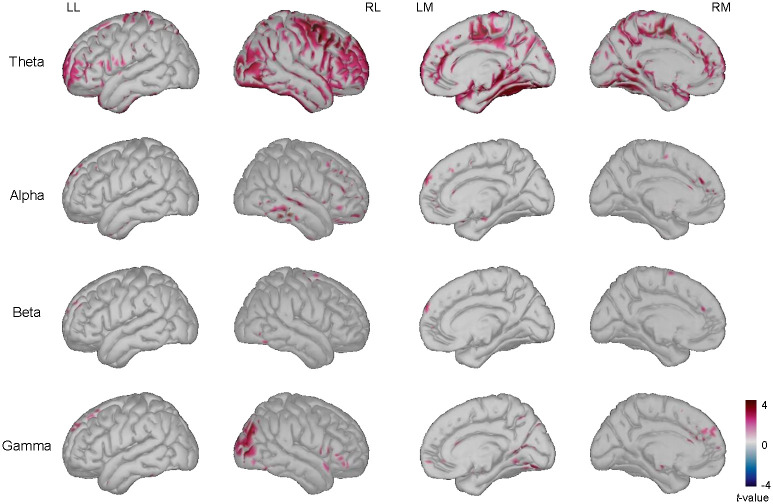
Significant difference between power for later remembered items vs. forgotten items during stimulus presentation for retrieval task. The *t*-values of the power over theta, alpha, beta, and gamma bands when comparing later remembered items to forgotten items at 700–1,000 ms during the presentation of the stimulus (two-tailed paired *t*-test, *p* < 0.05 with Bonferroni correction). LL = left lateral view; RL = right lateral view; LM = left medial view; RM = right medial view.

We also evaluated the changes in the decoding task for estimated source activity data; an increase in the theta, alpha, beta, and gamma band power was found over 700–1000 ms. A significant difference was found in the frontal, parietal, temporal, and occipital regions for the theta band; in the temporal region for the alpha band; and in the right occipital region for the gamma band. Additionally, significant differences of the theta band power data were identified in the medial parietal, precuneus, parahippocampal, and lingual regions. Regarding gamma band power, there was a difference in the lingual and medial prefrontal regions. Fig 7 shows the difference when comparing remember and forgotten trials for the decoding task during 700–1,000 ms.

### Classification performance

Table 1 shows results of pre-stimulus segments when using LDA and CNN models for different frequency bands and feature extraction methods. While Table 2 shows results of on-going segments. Since statistical differences were found at -300–0 ms and 400–800 ms, data points from these windows were used during classification. CNN outperformed LDA model for all cases. The highest value for surface potentials was obtained using the theta band power, 64.81 ± 5.23% and 63.43 ± 4.99% for pre-stimulus and on-going stimulus segments, respectively. The highest value for source activity was obtained using the alpha band power for pre-stimulus (67.15 ± 4.87%) and the gamma band power for on-going stimulus (67.15 ± 6.27%).

**Table 1:** AUC (%) for pre-stimulus segments.

Feature	Model	Frequency			
		Theta	Alpha	Beta	Gamma
Surface Potentials	LDA	54.71 ± 4.86	59.47 ± 5.46	60.30 ± 5.68	56.68 ± 4.62
	CNN	64.81 ± 5.23	63.02 ± 6.77	61.88 ± 3.97	61.37 ± 5.13
Source activity	LDA	54.96 ± 4.45	59.78 ± 6.38	59.00 ± 6.01	57.32 ± 6.31
	CNN	66.87 ± 6.04	67.15 ± 4.87	67.04 ± 6.31	67.09 ± 5.51

**Table 2:** AUC (%) for on-going stimulus segments.

Feature	Model	Frequency			
		Theta	Alpha	Beta	Gamma
Surface Potentials	LDA	55.45 ± 6.31	57.81 ± 6.63	60.57 ± 5.61	59.55 ± 4.89
	CNN	63.43 ± 4.99	62.31 ± 5.51	61.34 ± 4.55	61.17 ± 6.12
Source activity	LDA	54.60 ± 5.72	56.17 ± 5.68	64.93 ± 5.98	59.38 ± 4.76
	CNN	66.49 ± 3.97	66.91 ± 4.21	66.85 ± 4.87	67.15 ± 6.27

**Fig 8:**
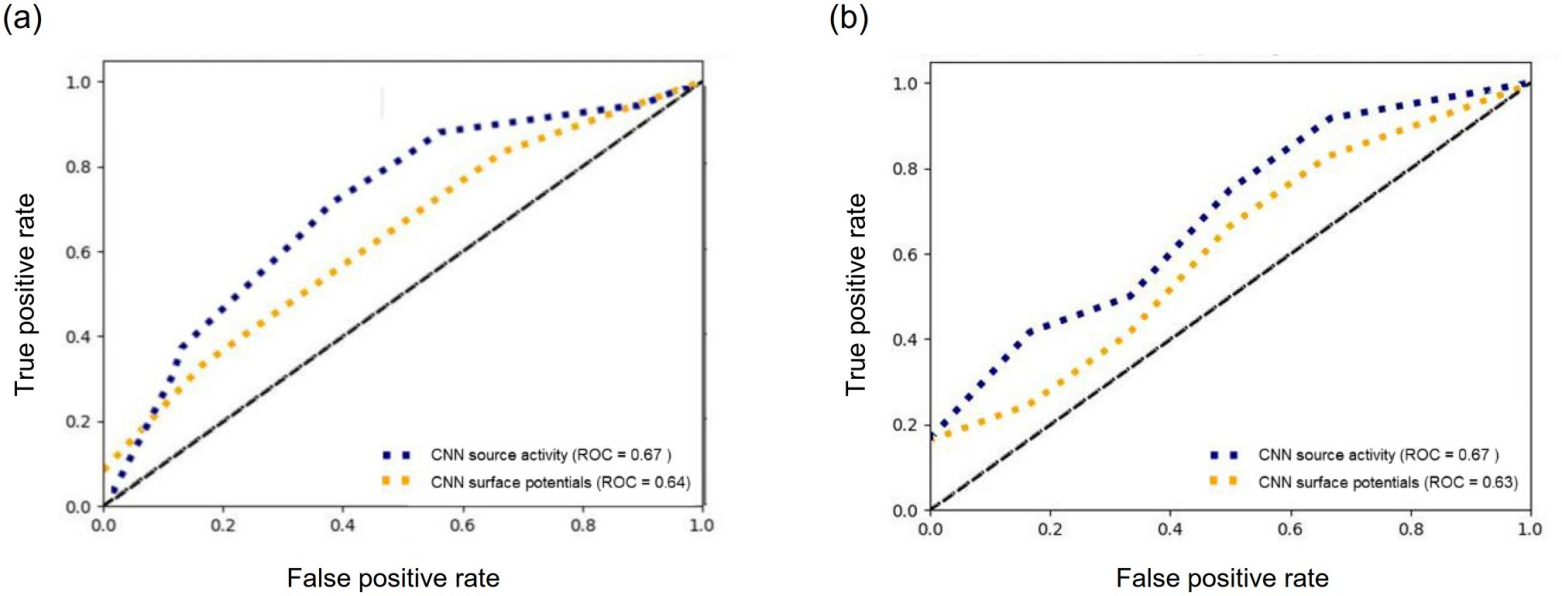
ROC AUCs (receiver operating characteristic area under curves for (a) pre-stimulus and (b) on-going stimulus segments. ROC curve was calculated when using source activity and surface potentials, for each case we use the frequency band that produce higher results.

Table 3 shows the statistical results of the ANOVA test for the CNN model. No significant difference was found when comparing prediction accuracies for different frequency bands. However, a significant difference was found when comparing the prediction accuracy of surface potentials and source activity for pre-stimulus and on-going stimulus (*F*_(1)_ = 11.48, *p* = 0.001; and *F*_(1)_ = 8.78, *p* = 0.003). Post-hoc test results showed that there was a difference when comparing classification accuracies of surface potentials and source activity for pre-stimulus segments in all frequencies (alpha band: *t*_(12)_ = 2.526, *p* = 0.015; beta band: *t*_(12)_ = 2.386, *p* = 0.034; gamma band: *t*_(12)_ = 2.422, *p* = 0.032) except for theta band (*t*_(12)_ = 1.108, *p* = 0.289). Additionally, there was significant difference in the alpha, beta and gamma band for on-going stimulus segments (*t*_(12)_ = 2.81, *p* = 0.011; *t*_(12)_ = 3.95, *p* = 0.002; *t*_(12)_ = 2.45, *p* = 0.032; respectively); however, there was no significant difference in theta band (*t*_(12)_ = 1.042, *p* = 0.182). Finally, there was no interaction effect between frequency and features for both pre and on-going stimulus segments during the encoding task. Fig 8 depicts the receiver operating characteristic curve for pre-stimulus and on-going stimulus segments.

**Table 3:** Statistical results for classification performance.

Factor	Pre-stimulus			On-going stimulus		
	*df*	*F*	*p*-value	*df*	*F*	*p*-value
Frequency	3	0.63	0.599	3	0.59	0.625
Feature	1	11.48	0.001	1	13.28	<0.001
Frequency × Feature	3	0.54	0.655	3	0.48	0.694

Hierarchical linear regression (HLR) has been used to analyze EEG signals and reveal the effects of different parameters of the model [41]. We used HLR to compare the two different feature extraction methods and specified surface potentials and spatial features (source activity) as the independent variables. Results showed that *R*^2^-values were higher for all subjects when including spatial features (see S1 Table). For pre-stimulus segments, average *R*^2^-values across all participants were 0.57 and 0.66 for surface potentials and spatial features (source activity) respectively. For on-going stimulus segments, average *R*^2^-values across all participants were 0.61 and 0.67 for surface potentials and spatial features (source activity) respectively. Statistical results showed that the changes in *R*^2^ and F-values were significant for pre-stimulus (*t*_(12)_ = -11.45, *p* < 0.001; *t*_(12)_ = -9.94, *p* < 0.001) and on-going stimulus (*t*_(12)_ = -5.83, *p* < 0.001; *t*_(12)_ = -6.11, *p* < 0.001).

**Fig 9:**
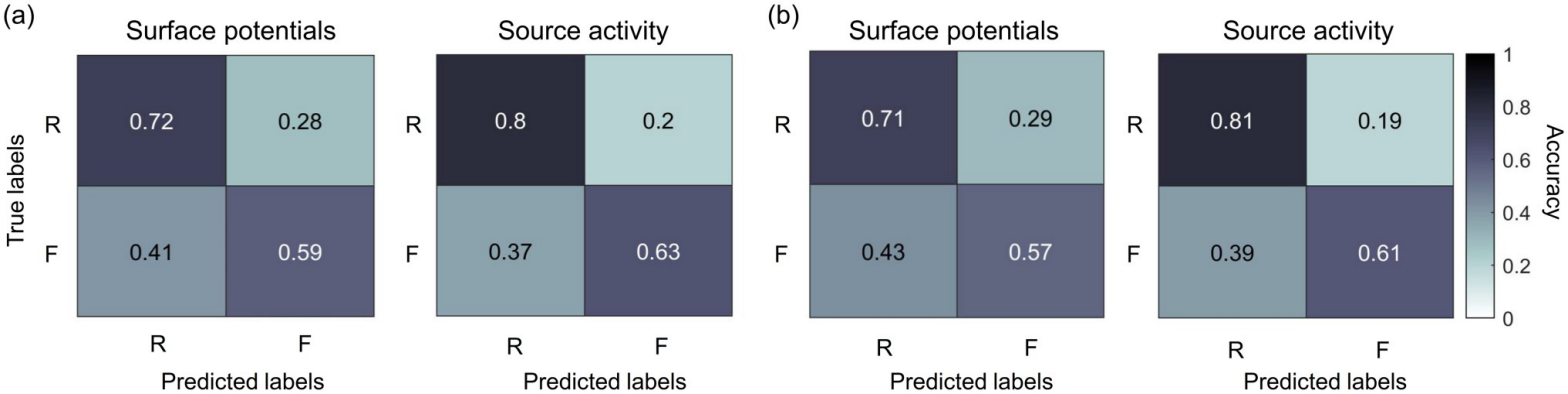
Normalized averaged confusion matrix for surface potentials and source activity when using CNN. (a) Confusion matrix for pre-stimulus segments and (b) on-going stimulus segments were presented. R = remembered items; F = forgotten items.

Fig 9 illustrates the normalized average confusion matrix for pre-stimulus and on-going segments. The model showed higher sensitivity than specificity, in other words, the percentage of later remembered samples classify correctly is higher than the later forgotten samples. This trend is present for both source activity and surface potentials, however, when comparing classification accuracies per class for different features extraction methods, source activity performed better.

## Discussion

Our results show that it is possible to predict retrieval task performance in a declarative memory task using the reconstructed source activity data with higher accuracy than using surface potentials. Changes in frequency bands have been used to identify later remembered or forgotten items presented during the encoding task [2]. Therefore, we evaluated the differences in classification performance using signals from four different frequency bands. The highest accuracy for the pre-stimulus segment was 67.15 ± 4.87% using the alpha band and 67.15 ± 6.27% for the on-going stimulus segment using the gamma band when both used source activity and the CNN model. There was no significant difference when compared with the results of other frequency bands. However, a significant difference was found when comparing the AUC of surface potentials and source activity. Moreover, during the hierarchical linear regression analysis, a statistical difference was found between the changes in *R*^2^ and F-values of surface potentials and spatial features. Therefore, adding spatial features explains a significant amount of unique variance above of the already explained by surface potentials.

Previous studies found changes in neural oscillations during the encoding task related to later remembered items. The decrease in alpha and beta power have been related to the retrieval of long term memory; this change in power reflects the reactivation of the sensory features of a memory trace; decreased power over frontal, parietal, and left temporal electrode sites was linked to object retrieval, and the level of decrease varied in relation to the retrieval performance [30]. Hanslmayr et al. [27] reported a difference in brain oscillations between semantic and non-semantic encoding of episodic memories. Increased theta band power was linked to successfully remembered items during the non-semantic task, whereas decreased alpha and beta band power was linked to the semantic task (over the left frontal and right occipital, and left frontal and parietal regions, respectively); therefore, it was concluded that decrease in alpha and beta band power was related to the processing of objects’ semantic features. Salari and Rose [13] found a significant difference in theta and beta band power over the frontal and temporal regions prior to the presentation of the stimulus; this was applied later to modulate the presentation of the stimulus (previous studies found a decrease in beta band power). In line with previous research, we found significant differences in the power spectra, especially a decrease in the alpha band power and an increase in the beta and gamma band power in the pre-stimulus period. For the on-going stimulus period, there was a decrease in the alpha power and an increase in the beta band power. Increases in the beta and gamma band power have been related to attention and short and long term memory [42, 43]. Gamma frequency synchronization in visual areas can promote synaptic changes in areas responsible for encoding long-term memory [44].

For the retrieval task, we found changes in neural oscillations, especially in theta and gamma bands. Many studies have reported an increase in gamma band power related to selective attention and visual perception [11]. In contrast, an increase in theta band power has been related to reflecting neuronal dynamics that are optimal for synaptic plasticity, which facilitates memory encoding [11]. Additionally, increased theta band power has been found to reflect mental effort [28].

Analysis of the source localization data resulted in the increase of power over the medial parietal areas, medial prefrontal, anterior and posterior cingulate regions, and a decrease over lingual and precuneus was present prior to the presentation of the stimuli related to successfully remembered items. Greater activation over frontoparietal regions, anterior and posterior middle frontal cortex, left frontopolar cortex, medial prefrontal cortex, and anterior cingulate cortex during successful retrieval has been previously reported [16]. In addition, the increase in theta and alpha power over medial temporal regions before the presentation of the stimulus has been suggested to reflect the activation of contextual information and preparatory processes [45]. In this regard, we found a similar pattern on the reconstructed signals using source localization that fMRI and ECoG studies related to memory process by estimating the source of cortical activity measured at the scalp [46]. These results show the importance of spatial features related to memory mechanisms.

We used signals from different frequency bands and performed classification using surface potentials and source activity. A significant difference was found in both cases; however, the highest results were obtained when using source activity data, especially when using a CNN network. This can be explained by the use of spatial features obtained through source localization. As mentioned before, previous ECoG and fMRI studies reported changes in subcortical structures [16, 45], which could provide additional information for classification. In other words, we used source localization to reconstruct the source of the cortical response, which provides spatial information. As a result, We achieve a 5% improvement in performance compared to the obtained performance using surface potentials, which was almost the same as classification performance with ECoG signals reported by previous studies. It has also been noticed that different frequency bands can provide different features and are related to different mechanisms involved in memory, such as attention and semantic processing. This is supported by the obtained results. The confusion matrix revealed that high sensitivity is present for both source activity and surface potential. Even though the specificity was higher than the chance level and increase for source activity, it is lower than the sensitivity. This could affect the future use of our method in memory-related applications. For example, if we were to apply our method to increase memory efficiency a high true negative rate is necessary since this could detect correctly the items that need be learned again. Similarly, for memory-related diagnosis disease, we could detect if the forgotten rate is within normal range or not.

One limitation of this study is the imbalance between remembered and forgotten trials; this can be explained by the nature of the task (usually remembered items are greater than forgotten ones). We tried to address this limitation using SMOTE. Another limitation is the type of stimulus provided; the task difficulty could have varied because stimuli were randomly selected from a pool of the most commonly used words. Additionally, in this study, participants were 24–31 years old with no known memory-related problems. It is intuitive to assume that depending on the age group to be analyzed; features related to the memory process could change, therefore we can not generalize our findings to older age groups. In future work, we will apply our method to data from older adults and test whether classification is possible or not and whether source localization could increase performance. We decided to further implement other classification methods in order to increase the true negative rate, as mentioned before this has an important role for future use in memory-related applications. Furthermore, methods for better source estimation from surface potentials need to be studied. Finding optimal activity sources would directly help improve performance.

## Conclusion

Our study investigated the prediction of successful item recall using reconstructed activity through source localization during declarative memory, which can be used to enhance learning efficiency and diagnose and treat diseases that impair memory abilities [1]. Enhancement of memory abilities has been attempted before. Previous studies used current or magnetic stimulation or neurofeedback to modulate brain activity [47, 48]; however, the effects of such approaches have varied from study to study, and it is hard to measure their effectiveness. Our study proposed the possibility that depending on the brain state of when the stimulus is presented, that stimulus is more likely to be remembered or forgotten [49]. Our findings can also help to explore the neural correlates of the memory process by proposing the use of spatial information through source localization. Moreover, the prediction of successful item recall was studied in working memory paradigms and a higher prediction accuracy was achieved due to the nature of the task. Therefore, it is necessary to prove our approach’s validity in different memory paradigms and improve the prediction accuracy.

## Supporting information

S1 Fig ERP at Cz over all subjects for a) encoding and b) decoding task. dGFP was calculated for all channels for c) encoding and d) decoding task (amplitude: *μ*V and time: ms). Statistical analyses revealed significant differences between remembered and forgotten items at different time points. Additionally, the dGFP coefficients showed a high signal-to-noise ratio.
(TIF)

S1 Table *R*^2^ and F-values for pre-stimulus and on-going stimulus segments. Hierarchical linear regression was computed to compared the effects of using surface potentials alone or including spatial features trough source localization.
(TIF)
