# Supplementary figures and images for: Decoding declarative memory process for predicting memory retrieval based on source localization
Source: PLoS One. 2022 Sep 8;17(9):e0274101. doi: 10.1371/journal.pone.0274101 (PMC9455842; doi:10.1371/journal.pone.0274101)

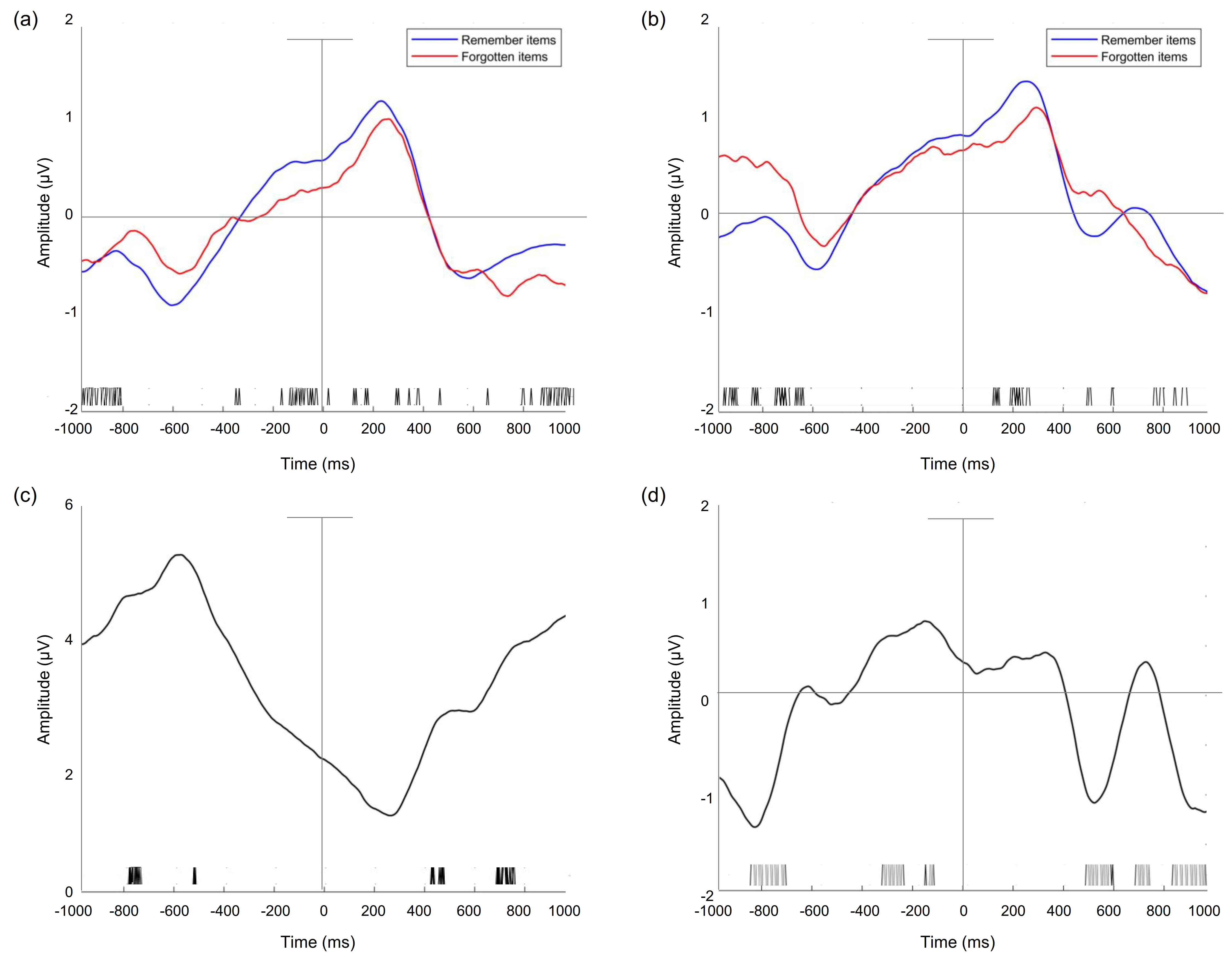

Supplement: S1 Fig — dGFP was calculated for all channels for c) encoding and d) decoding task (amplitude: μV and time: ms). Statistical analyses revealed significant differences between remembered and forgotten items at different time points. Additionally, the dGFP coefficients showed a high signal-to-noise ratio. (TIF) [file pone.0274101.s001.tif]

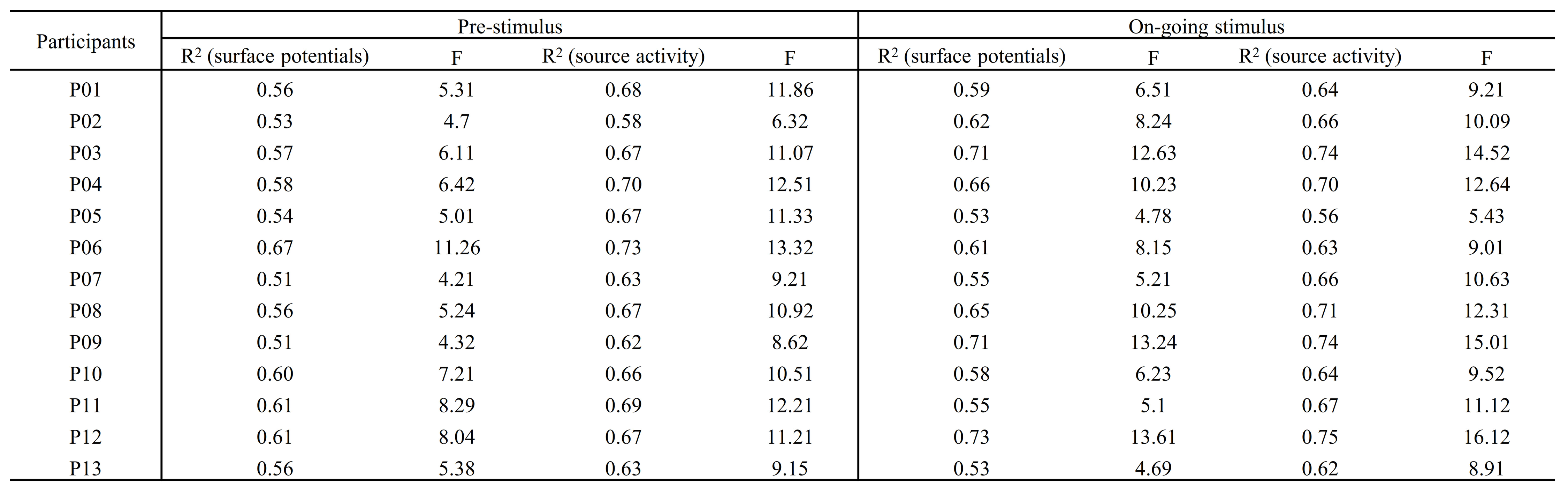

Supplement: S1 Table — Hierarchical linear regression was computed to compared the effects of using surface potentials alone or including spatial features trough source localization. (TIF) [file pone.0274101.s002.tif]
